# Acute Effects of Beetroot Juice Supplementation on Isometric Muscle Strength, Rate of Torque Development and Isometric Endurance in Young Adult Men and Women: A Randomized, Double-Blind, Controlled Cross-Over Pilot Study

**DOI:** 10.3390/nu14224759

**Published:** 2022-11-10

**Authors:** David Poredoš, Zala Jenko Pražnikar, Žiga Kozinc

**Affiliations:** 1 Faculty of Health Sciences, University of Primorska, Polje 42, SI-6310 Izola, Slovenia; 2 Andrej Marušič Institute, University of Primorska, Muzejski trg 2, SI-6000 Koper, Slovenia

**Keywords:** beetroot, knee extension, maximum voluntary contraction, strength endurance, nitrates

## Abstract

This study was conducted to investigate the effect of concentrated beetroot juice on isometric strength and knee extensor muscle endurance in healthy adults. We conducted a randomized cross-over, double-blind experiment in which participants (18 healthy, physically active adults, 9 men, 9 women) consumed either concentrated beetroot juice (140 mL) or low-nitrate control supplement 2.5 h before the measurement. Isometric maximum strength (peak torque), explosive strength (isometric rate of torque development), and strength endurance at 50% of peak torque were measured on an isometric dynamometer. The results showed that concentrated beetroot juice had no effect on the maximum voluntary isometric strength and rate of torque development of the knee extensors. The only exception was the maximum rate of torque development, for which a positive influence was demonstrated only in men. As for the endurance of the knee extensors, the supplement had a positive effect in men (endurance time increased from 86.4 ± 46.1 s to 103.4 ± 53.7 s; *p* = 0.022), but not in women. The absence of effect on maximal voluntary strength is consistent with previous research. One the other hand, improvements in endurance and rate of torque development in men only point to an important aspect of a previously under-researched area of sex-specific responses to nitrate supplementation.

## 1. Introduction

Nitric oxide (NO) is a signaling molecule synthesized by two pathways, either from L-arginine (oxygen dependent pathway) [1] or from nitrites previously reduced from nitrates [2]. The amount of available NO can be influenced by the consumption of foods rich in nitrates (NO_3_^−^) [3], such as beetroot [4].

Over the years, the use of beetroot juice has increased, especially among endurance athletes [5]. Beetroot juice concentrate (BJC) dietary supplements are a safe, affordable and reliable source of inorganic nitrates, which is particularly useful in countries where the sale of sodium nitrate is not allowed [6]. The International Olympic Committee has recently classified dietary supplements containing high levels of nitrate, along with creatine and caffeine, as performance enhancing supplements [7]. Previous studies using BJC supplements have reported improved time to exhaustion during endurance exercise, improvements in the time trials lasting up to 40 min, improvement in contractile function of type II muscle fibers during high intensity exercise and improvements in recovery during intermittent efforts [8]. Nitrate supplements are typically taken 2 to 3 h before physical activity in the form of the BJC and/or sodium nitrate, prescribed in absolute and relative amounts of 300 to 600 mg [9] or 0.1 mmol/kg of body weight per day [10] and up to 10 mg/kg of body weight [11].

Production of NO is facilitated in low pH and low oxygen levels environments that occur in skeletal muscle during intense exercise [8]. NO acts as a vasodilator and triggers physiological responses such as increased muscle blood flow and decreased oxygen cost during submaximal exercise [12]. In addition, NO can improve glucose uptake and mitochondrial efficiency [13]. The improvements in contractile function, which are most evident in fast muscle fibers (type II) [14], are likely underpinned by improved efficiency in the release and subsequent re-uptake of calcium from the sarcoplasmic reticulum [15], which may also improve force-generating capacity of muscle fibers [16]. The beneficial effects of nitrate supplementation in continuous endurance exercise and high-intensity interval exercise have been established in several previous studies [8], while there is a lack of empirical studies on the contractile properties on a local muscular level. The studies that investigated the effect of nitrates on maximal strength (maximal force/torque-generating capacity) found no effect on maximal isometric knee torque [17,18,19,20], nor hand grip force [21,22,23]. A positive effect on isometric mid-thigh pull force was observed in one study [24]. In addition, a positive effect of nitrate supplementation on isokinetic knee torque measurements is evident, but only at higher angular velocities [25]. The rate of torque development (RTD), a measure of explosive strength, has been poorly studied. Previous studies have indicated that RTD may be a stronger predictor of athletic performance [26] and injury rehabilitation [27] than maximal strength. The increase in sarcoplasmic reticulum calcium release and re-uptake observed in rat muscle fibers [15] suggests that nitrate supplementation may have an effect on RTD [28]. To date, two studies have examined chronic (7-day) nitrate supplementation on knee extensor RTD and found either no effect [29], or an increase in RTD only in the fatigued state [20].

While several studies have investigated the effects of nitrates on local muscle endurance during dynamic tasks and produced mixed results [19,30,31], there is a dearth of research on the effects of nitrates on isometric endurance. One study examined the effect of sodium nitrate on forearm muscle isometric torque endurance and found no effect [32]. Other studies have examined muscular endurance during intermittent isometric efforts [21,23], however, it is currently unknown whether nitrate supplementation can improve endurance during sustained isometric efforts. Given that isometric contractions restrict blood flow [33], nitrate supplementation could be particularly effective in boosting recovery between repeated sustained isometric efforts. In addition, a majority of studies investigating the effect of nitrate supplementation on muscle strength has included only men [19,21,22,23,24,29,31] or did not perform comparisons between men and women [30]. Therefore, further investigation of sex-specific responses to nitrate supplementation is needed. In particular, women tend to show lower susceptibility to muscular fatigue [34], which is likely explained by larger proportion of oxidative fibers [35], greater muscle capillarization [36] and larger intra-muscular triglyceride stores [37]. Thus, it should be investigated if man and women benefit from nitrate supplementation to the same extent when it comes to mitigating isometric fatigue. 

The purpose of this study was to investigate the acute effect of nitrate supplementation (in the form of BJC) on isometric maximal strength (peak torque), explosive strength (RTD) and local endurance during sustained isometric contraction for the knee extensor muscle group. RTD is one of the most important determinants of neuromuscular ability and athletic performance in general [38,39], while sustained isometric efforts are also present in many sports, such as gymnastics, martial arts and surface water sports. We hypothesized that BJC would improve RTD and isometric endurance, and in particular, the recovery between subsequent isometric efforts. We also hypothesized that BJC will have no effect on maximal strength.

## 2. Materials and Methods

### 2.1. Participants 

For this study, we used a convenience sample, whereby the participants were recruited through advertisement on the faculty’s website and social media, and word of mouth. Initially, we enrolled 22 healthy volunteers with no known cardio-vascular, metabolic neurological or musculoskeletal conditions. Inclusion criteria were age older than 18 years and being at least moderately physical active. Other exclusion criteria were pregnancy, breastfeeding, and smoking or taking medications (especially antacids and proton pump inhibitors) in the past 3 months. Participants were instructed to refrain from taking stimulants (caffeine, pre-workout supplements) for 12 h and from consuming alcohol and nitrate-containing foods for 24 h before coming to the laboratory. The detailed list of nitrate-rich foods was provided in advance. In addition, participants were asked to refrain from brushing their teeth, using mouthwash, and chewing gum on the day of their visit to the laboratory. Finally, they were asked not to change their dietary habits during the course of the study. The experiment was reviewed approved by the Republic of Slovenia’s Medical Ethics Committee of the (approval no.: 0120-690/2017/8) and was performed in accordance to the latest revision of Declaration of Helsinki. The protocol was registered in CliniclTrials.gov system (ID: NCT05567926).

### 2.2. Study Design

**Figure 1:**
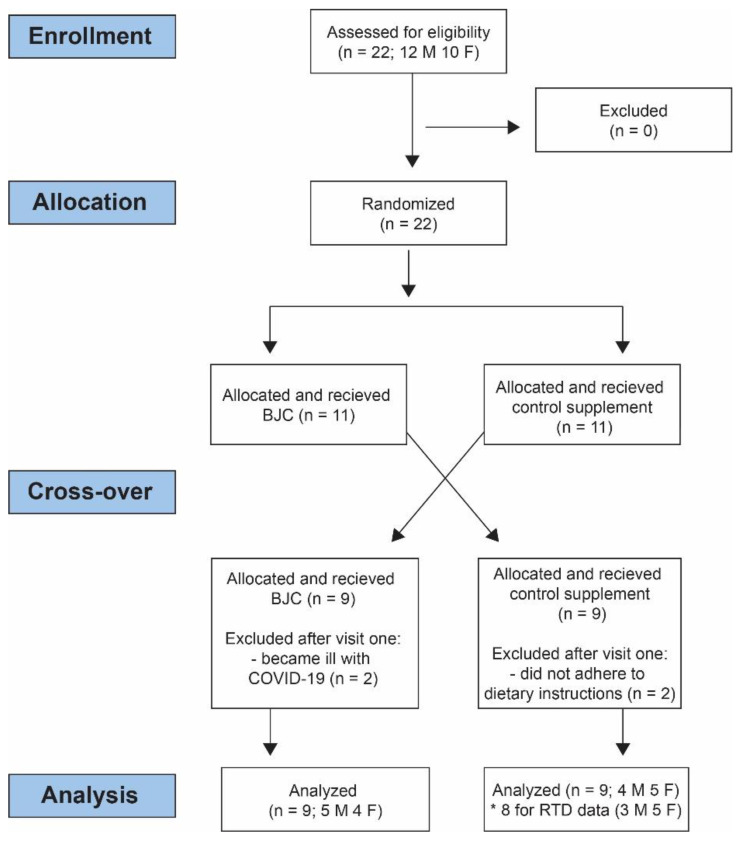
Study flowchart. * One participant excluded due to poor signal quality.

This pilot study was conducted as a cross-over, randomized, double-blind controlled trial. The crossover intervention study lasted 8 days and was divided into 2 different intervention days separated by a 7-day period. To minimize within-subject variation, the study was designed so that both sessions were conducted at the same time of day (±1 h). A blinded investigator randomly assigned participants to one of the two treatment orders. Randomization with stratification was performed using the free open-source desktop application MinimPy [40]. The stratified variables were sex and supplement; we used two randomly assigned separate sequences for men and women, and two randomly assigned separate sequences for condition (control; experimental). During the first session, 11 participants consumed 140 mL of BJC, whereas 11 participants consumed 140 mL of low-nitrate control supplement 2.5 h before the measurements. During the second session, participants consumed the alternative beverage. Both supplements were provided in black opaque jars. The study was randomized and blinded to participants and clinical investigators. The flowchart detailing the study design is shown in Figure 1.

### 2.3. Beetroot Juice Supplement and Low-Nitrate Control Supplement

In the experimental condition, participants consumed 140 mL of BJC (2 × 70 mL Beet-It-Pro Elite Shot, James White Drinks Ltd., Ipswich, UK) containing 12.9 mmol or 800 mg of nitrates. The low-nitrate control supplement consisted of 140 mL blackcurrant juice to which sugar and lemon juice were added to match the carbohydrate content and flavor of BJC. Thus, both beverages contained the same amount of carbohydrates and sugar, while BJC contained, in addition to nitrates, an additional 5 g of proteins and a correspondingly higher energy value. The details of the nutrient composition can be found in Table 1. Both the experimental and low-nitrate control supplement drinks resembled each other in color and size to ensure that they were indistinguishable.

### 2.4. Measurement Protocol

**Figure 2:**
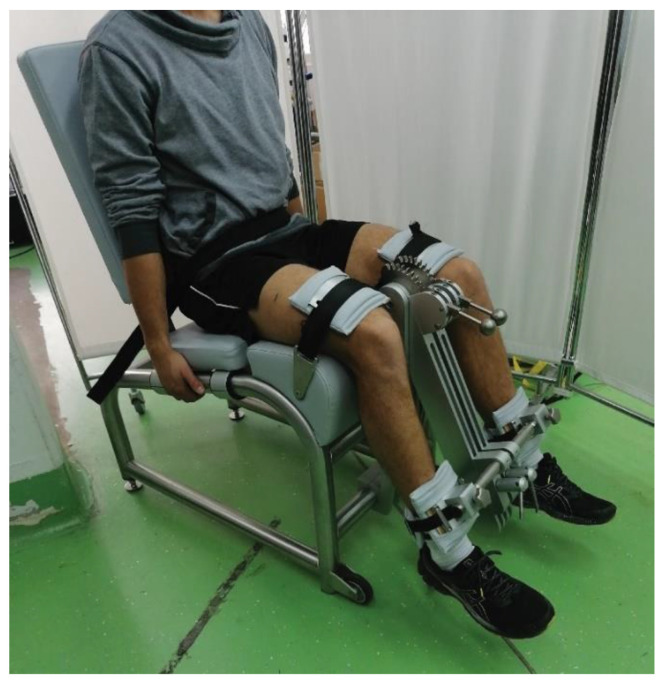
Set-up for maximal strength and endurance measurements.

Prior to muscle strength and endurance measurements, a warm-up was performed (consisting of 10 min of light-intensity running), 10 squats, 10 lunges, and single-leg deadlifts. All measurements were performed on an isometric dynamometer specifically designed to measure knee torque (S2P, Ljubljana, Slovenia). The sampling rate was set at 1000 Hz. The setup is shown in Figure 2. The excellent reliability of this dynamometer has been reported previously [41]. We examined only the right leg in all participants. The hip and knee angles were set at 90° and 60°, respectively, which was checked with a manual goniometer. First, three warm-up repetitions were performed at 50%, 75%, and 90% of the perceived maximal effort. Then, 3 repetitions of maximal voluntary contraction of knee extension were performed, with 1 min rest in between. Participants were instructed to contract “as fast and as hard as possible” and maintain maximal effort for ~3–4 s [42] which allowed quantification of peak torque and rate of torque development. Participants received visual feedback on the torque-time trace and were verbally encouraged during all repetitions. After maximal voluntary contraction measurements, we also assessed knee extensor endurance. The target torque was set at 50% of the previously achieved peak torque. Participants were shown the horizontal line on the screen, which served as a reference. They were instructed to keep the torque just above this value for as long as possible. The measurement was terminated when the participant could not maintain the target torque for more than 2 s. After a 2-min rest, another isometric endurance measurement with the same target torque was performed to assess if BJC supplementation would have any effect on recovery between the repetitions. 

### 2.5. Data Analysis

The data were automatically processed in the custom-made software (ARS Dynamometry, S2P, Ljubljana, Slovenia. Maximal strength was evaluated by calculating peak torque during maximal voluntary contraction, after applying a 1-s moving average filter. Explosive strength was assessed as RTD (the first derivative of torque with respect to time). In addition to peak RTD, we also assessed RTD at 0–50, 0–100 and 0–200 ms time windows. The onset of contraction was determined manually. The average of the best two repetitions was considered for further analysis. Isometric endurance was defined as the total time during which the torque level was kept above target (50%). 

### 2.6. Statistical Analysis

Data are presented as means and standard deviations. Differences between experimental and control conditions for peak torque and RTD were assessed with 2-way mixed model analysis of variance, with condition as within-subject factor and sex as between-subject factor. Post-hoc t-tests with Bonferroni correction were performed when appropriate. For the muscle endurance, we used the 3-way analysis of variance, with repetition with an additional within-subject factor. Effect sizes for main effects were expressed as partial eta-squared (η^2^) and interpreted as trivial (<0.02), small (0.02–0.13), moderate (0.14–0.26) and large (>0.26) [43]. For the *t*-test, we calculated the Cohen’s d, which can be interpreted as trivial (< 0.2), small (0.2–0.5), moderate (0.5–0.8), large (0.8–1.2) and very large (>1.2) [44]. We also assessed the intra-visit reliability with intra-class correlation coefficient (ICC; two-way random, single measures, absolute agreement). We interpreted the ICC values < 0.5 as poor reliability, values between 0.5 and 0.749 as moderate reliability, values between 0.75 and 0.899 as good reliability, and values equal or greater than 0.90 as excellent reliability [45]. All analyses were carried out using SPSS statistical software (IMB, Armonk, NY, USA). The threshold for statistical significance was set at α < 0.05.

## 3. Results

Initially, 22 participants were enrolled. Two participants dropped out after only one measurement because they became ill with COVID-19. In addition, two participants were excluded because they did not strictly adhere to the dietary guidelines provided by the investigators. Thus, the final sample consisted of 18 participants (9 males, 9 females), aged 29.1 ± 6.6 years, with a body mass of 74.7 ± 15.3 kg and a body height of 174.4 ± 10.9 cm. The participants reported to be moderately physically active, performing on average 3 sessions of low-to-moderate intensity endurance exercise/physical activity (range 1–5) per week, while none of the participants performed regular resistance exercise. None were competitive athletes in the past. In addition, none of the participants reported consuming any nutritional supplements in the past 6 months. RTD data from one additional participant were excluded due to errors in the signal observed at one visit. In the experimental condition, the nitrate content therefore ranged from 7.7 to 15.1 mg/kg of body mass, corresponding to 124.1 to 243.4 µmol/kg. Reliability for peak torque was excellent for both visits (ICC = 0.98–0.99). Reliability for RTD measurements was good to excellent (ICC = 0.81–0.94 for the experimental condition; ICC = 0.87–0.94 for the control condition).

### 3.1. Maximal Strength and RTD

**Figure 3:**
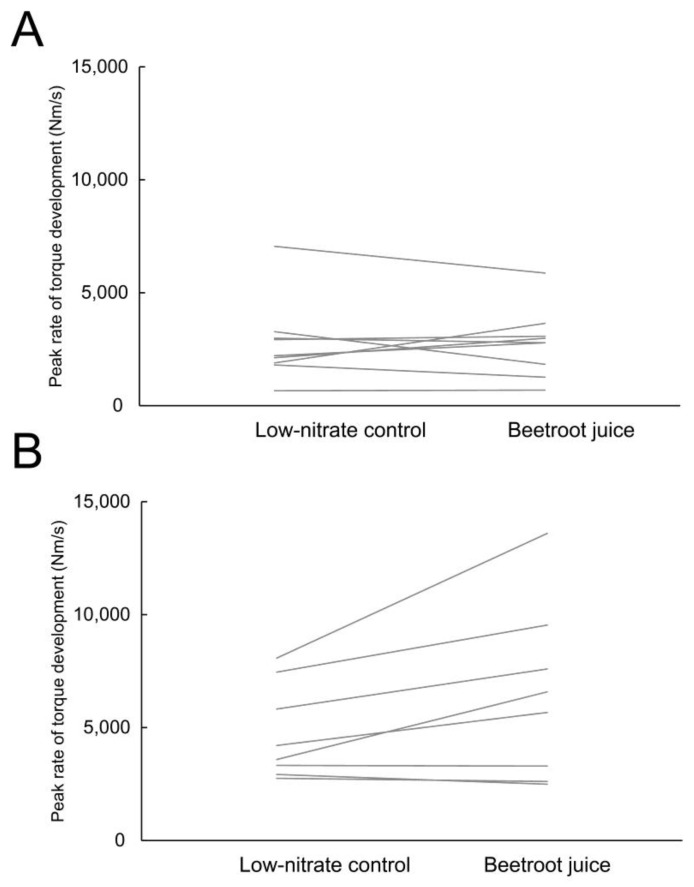
Individual responses regarding peak rate of torque development for female (**A**) and male (**B**) participants.

The descriptive statistics and main effects for peak torque and RTD results are shown in Table 2. Peak torque (*p* < 0.001; η^2^ = 0.64) and all RTD variables (*p* = 0.001–0.023; η^2^ = 0.30–0.64) were higher in males than females. There were no interactions between sex and condition (*p* = 0.075–0.530), nor the main effects of condition (*p* = 0.096–0.737). The exception was peak RTD, for which there was a statistically significant interaction (*p* = 0.041; η^2^ = 0.25) and main effect of condition (*p* = 0.042; η^2^ = 0.25). Post-hoc *t*-test for peak RTD revealed no effect of condition in females (*p* = 0.998; mean difference = −0.2 Nm/s), however, BJC moderately and statistically significantly increased peak RTD in males (BJC: 6421 ± 3835 Nm/s; control: 4766 ± 2090 Nm/s; *p* = 0.048; d = 0.53). The results for peak RTD are shown in Figure 3.

### 3.2. Endurance 

**Figure 4:**
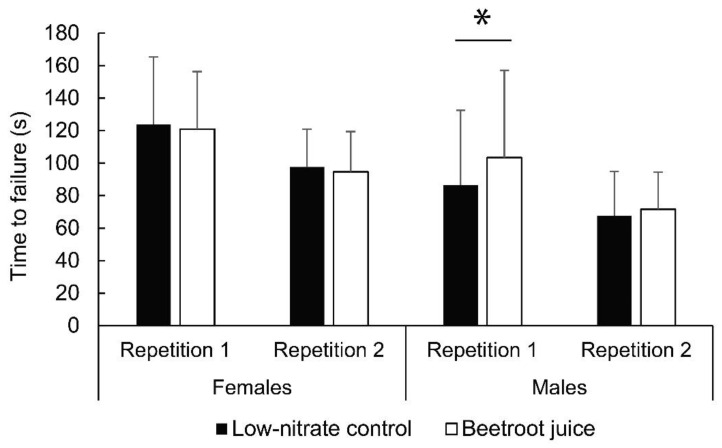
Results regarding muscle endurance (time to failure). * Statistically significant difference at *p* < 0.05.

For endurance (time to failure), we found no sex × condition × repetition interactions (*p* ≤ 0.212). However, there was a statistically significant interaction between sex and condition (*p* = 0.047; η^2^ = 0.24), indicating a different response to BJC supplementation in males and females. At the first repetition, males increased their time to failure from 86.4 ± 46.1 s (control) to 103.4 ± 53.7 s (BJC) (*p* = 0.022; d = 0.37). No differences were observed between conditions at the second repetition (*p* = 0.355) or for female participants in general (*p* < 0.675). The results are shown in Figure 4.

## 4. Discussion

The purpose of this study was to investigate the acute effect of nitrate (BJC) supplementation on knee extensor isometric torque, explosive strength (RTD) and local endurance. According to our hypothesis, maximal isometric torque was not affected by nitrate supplementation. As for explosive force, only peak RTD was increased in men. Finally, local muscle endurance was also increased only in males. However, contrary to our hypothesis, the effect on endurance was seen only in the first repetition. These results suggest that acute nitrate supplementation could improve isometric RTD and local isometric endurance in physically active men.

In our study, BJC intake had no statistically significant effect on maximal torque (BJC: 256.5 ± 76.3 Nm, control: 260.9 ± 88.4 Nm), which is consistent with the results of previous research. Studies that measured the effect of beetroot juice (0.5 L of juice containing 10.2 mmol of nitrates) at different time of ingestion (acute 2.5 h before the measurement, ingestion of the supplement for 5 or 15 days) did not detect the effect on maximal voluntary isometric strength [17]. Likewise, Tillin et al. [20] also reported that BJC had no effect on knee extensor torque in fatigued and non-fatigued conditions. There were also no statistically significant differences in isometric knee extensor torque after taking BJC for 3 to 6 days [18,19,46]. Accordingly, two very recent meta-analyses concluded that BJC supplementation has no effect on isometric muscle strength [25,47].

We found no statistically significant effect of BJC on RTD outcomes, except for peak RTD, for which the BJC had an enhancing effect only in men. Accordingly, a study examining chronic BJC supplementation [29] found no effect on RTD at different time intervals. However, they reported moderate effects on evoked force and RTD during evoked contractions, demonstrating that BJC affects intrinsic muscle contractile function. While the underlying mechanisms of this effect are not fully understood, others have suggested an increase in myoplasmic calcium concentration or increased or altered sensitivity of cross-bridges to calcium as possible candidates [15,29]. However, RTD during voluntary contractions (as used in this study) is also largely underpinned by neural factors, predominantly the rate of motor unit recruitment and, to some extent, motor unit firing rates [48]. The variability of these neural determinants between participants inevitably leads to higher variability in RTD results, making possible effects of BJC difficult to detect. The finding of increased peak RTD in males and a similar trend for RTD at 50 ms (main effect of condition, *p* = 0.096) suggest that the effects of BJC could be detected with larger samples or bypassing the neural determinants of RTD (i.e., assessment of evoked contractions).

There is a lack of research on sex-specific effects on maximal strength and RTD. A recent systematic literature review and meta-analysis of randomized controlled trials [2] found a statistically significant effect of nitrates on endurance time trials time and time to exhaustion during continuous and intermittent tasks, but these effects could not be confirmed when only female participants were considered. One explanation for these differences could be a higher proportion of slow-twitch muscle fiber in women [35], as nitrate supplementation mainly has an ergogenic effect on fast-twitch muscle fibers [8,15]. On the other hand, women have higher endothelium-dependent dilation [49], which could lead to higher plasma nitrite concentrations due to increased oral activity of nitrate-reducing bacteria [50]. Estrogen also plays a role in increased expression of the eNOS gene, the product of which plays an important role in the release of NO from the endothelium [51]. Previous studies have also reported results that contradict ours. For example, BJC was found to have a greater effect on isokinetic knee strength performance in women compared with men [52]. An important limitation of this study is that we did not account for menstrual cycle and dietary nitrate intake. Women tend to consume more vegetables than men [53], and although no nitrate-rich foods were consumed 24 h prior to the experiment, greater chronic intake of nitrates could attenuate the potential of BJC to improve muscle function [29]. A recent review highlighted several other sex-specific factors that need to be explored in the future, such as blood pressure responses, mitochondrial function after nitrate supplementation and skeletal muscle capacity to store nitrate [54].

Regarding isometric torque endurance, we found a positive effect in men (control: 86.4 ± 46.1; BJC: 103.4 ± 53.7 s). This could be again explained by the higher proportion of fast-twitch fibers (which are more sensitive to nitrate supplementation) [35] and lower muscle capitalization in men [36]. In existing literature, there is a lack of studies on the effect of nitrates on isometric endurance. Gasier et al. [32] observed no effect of sodium nitrate on forearm muscle endurance during isometric contraction in recreationally active subjects. In contrast, BJC supplements have an ergogenic effect during high-intensity intermittent dynamic tasks [16]. In our case, an increase in calcium release and re-uptake in the sarcoplasmic reticulum could improve the efficiency of excitation-contraction coupling, which could reduce the perceived effort for the same submaximal load. Indeed, perceived effort has been shown to be reduced during short, high-intensity tasks following BJC supplementation [55]. In addition, NO facilitates vasodilation, helping to maintain oxygen delivery to muscle fibers [56]. In the aforementioned study by Gasier et al. [32] no effect of sodium nitrate on blood flow during intermittent isometric forearm contractions was observed; however, it should be noted that their study was conducted under hypoxic conditions. There is evidence of possible effects of BJC supplements on motor unit recruitment during sustained isometric contractions [57]. 

Contrary to our hypothesis, the effect on BJC on isometric endurance was seen only during the first repetition. We expected that BJC could attenuate the recovery between the two isometric efforts via increased blood flow and oxygenation. However, it seems that blood flow is not a limiting factor in repeated isometric force production [33]. In addition, although isometric contraction restricts blood flow, nitric oxide and other vasoactive substances (e.g., adenosine) are released during contraction to dilate the blood vessels [58,59,60]. Thus, it could be that automatic response by the body is sufficient to cause a quick recovery of isometric force after fatigue, at least at the intensity used in this study (as indicated by similar values in both repetitions). Further research should examine the effects of BJC on isometric endurance and recovery at different contraction intensities. 

In addition to not considering the female menstrual cycle and habitual dietary intake of nitrates, the limitation of our study was that we did not use nitrate-depleted concentrated beetroot juice as a placebo in our study. In that case, both drinks would be comparable in terms of nutrient composition and bioactive compounds, differing only in the concentration of nitrates. It must be acknowledged that black currant itself could have some ergogenic effects, especially due to anthocyanins [61]. In addition, a true placebo (nitrate-depleted beetroot juice) would eliminate the possibility that the subjects recognize or discover the difference between liquids. Nitrate intake via concentrated beetroot juice was the same in all subjects and in some cases higher than the general recommendations. In addition, the effect of BJC on plasma NO or nitrite/nitrate concentration was not verified. However, since equal and smaller doses were previously shown to significantly increase plasma NO [12], we are confident that our BJC supplement was sufficient to achieve increases in plasma NO. Lastly, our sample size was small, particularly with respect to the comparison between men and women. Therefore, further studies will be needed to investigate the sex-specific effects of BJC on isometric muscle strength and endurance. 

## 5. Conclusions

This study demonstrated a beneficial effect of acute BJC supplementation on local muscle strength endurance in men, but not in women. In addition, potential effects on explosive strength (RTD) were also noted in males. This implies that male athletes who participate in sports that require sustained isometric contractions (e.g., gymnastics) may consider supplementation with BJC to enhance their performance. Further research is needed to elucidate the mechanisms underlying the observed effects and, in particular, to investigate why men and women seem to respond differently to BJC supplements. 

## Figures and Tables

**Table 1:** Nutritional details of beetroot juice and low-nitrate control supplement.

	Beetroot Juice	Low-Nitrate Control Supplement
Volume (mL)	140	140
Energy (kJ/kcal)	515/123	435/104
Carbohydrates (g)	25.2	25.2
Sugars (g)	23.8	23.5
Fats (g)	0	0
Saturated fats	0	0
Protein (g)	5.2	0.3
Sodium (g)	0.672	0.0028
Nitrates (mg)	800	not specified

**Table 2:** Descriptive statistics and main effects for peak torque and rate of torque developments outcomes.

	Descriptive Statistics				ANOVA					
	Women		Men		Sex		Condition		Interaction	
	BJC	Control	BJC	Control	*p*	η^2^	*p*	η^2^	*p*	η^2^
PT (Nm)	208.7 ± 61.9	188.6 ± 28.4	304.4 ± 58.2	333.3 ± 63.2	0.000	0.64	0.737	0.01	0.075	0.19
RTD50 (Nm/s)	1289.7 ± 546.3	1200.1 ± 460.2	2205 ± 866	2015.8 ± 925	0.022	0.30	0.096	0.17	0.535	0.03
RTD100 (Nm/s)	1166.1 ± 436.1	1014 ± 280.6	1827.5 ± 369.1	1730.1 ± 573	0.002	0.49	0.219	0.10	0.782	0.01
RTD200 (Nm/s)	782.6 ± 255	709.6 ± 149.3	1199.8 ± 239.5	1214 ± 268	0.000	0.58	0.540	0.03	0.366	0.06
RTDmax (Nm/s)	2766.4 ± 1500	2766.6 ± 1786.8	6421.3 ± 3836	4766.3 ± 2090	0.023	0.30	0.042	0.25	0.041	0.25

PT—peak torque; RTD—rate of torque development; BJC—beetroot juice concentrate.

## Data Availability

Not applicable.
